# A Q-methodology study of flare help-seeking behaviours and different experiences of daily life in rheumatoid arthritis

**DOI:** 10.1186/1471-2474-15-364

**Published:** 2014-11-01

**Authors:** Caroline A Flurey, Marianne Morris, Jon Pollock, Pamela Richards, Rodney Hughes, Sarah Hewlett

**Affiliations:** University of the West of England, Bristol, UK; University of Bristol, Bristol, UK; Ashford & St Peter’s Hospital Foundation Trust, Chertsey, UK; Academic Rheumatology Unit, The Courtyard, Bristol Royal Infirmary, Bristol, BS2 8HW UK

**Keywords:** Rheumatoid arthritis, Psychological adjustment, Psychological adaptation, Health care seeking behaviour, Help-seeking behaviour, Q-methodology, Mixed-methods, Qualitative

## Abstract

**Background:**

Previous studies have not addressed rheumatoid arthritis (RA) patients’ help-seeking behaviours for RA flares, and only one small qualitative study has addressed how patients experience daily life on current treatment regimes. Thus, this study aims to identify clusters of opinion related to RA patients’ experiences of daily life on current treatments, and their help-seeking behaviours for RA flares.

**Methods:**

Using Q-methodology (a methodology using qualitative and quantitative methods to sort people according to subjective experience), two separate studies were conducted with the same sample of RA patients (mean age 55, 73% female). Thirty participants sorted 39 statements about daily life (Q-study 1) and 29 participants separately sorted 23 statements about flare help-seeking (Q-study 2). Data were examined using Q-factor analysis.

**Results:**

Daily life with RA (Q-study 1): Three factors relating to the experience of living with RA were extracted and explained. Patients belonging to Factor A (mean age 62, 86% female) use effective self-management techniques to control the daily impact of RA. Those in Factor B (mean age 55, 75% male) struggle to self-manage and cope. Whilst patients in Factor C (mean age 42, 100% female) prioritise life responsibilities over their RA, reporting less impact.

Flare help-seeking (Q-study 2): Two factors explaining the experience of flare help-seeking (unrelated to the factors from Q-study 1) were extracted and explained. Factor X (68.8% on biologics) reported seeking help quickly, believing the medical team is there to help. Factor Y (0% on biologics) delay help-seeking, concerned about wasting the rheumatologist’s time, believing they should manage alone. All participants agreed they sought help due to intense pain and persistent, unmanageable symptoms.

**Conclusions:**

Patients with different characteristics appear to manage RA life in different ways and men may struggle more than women. Whilst all patients are prompted to seek help by persistent, unmanageable symptoms, some delay help-seeking. Further research is needed to quantify the severity of daily symptoms, the level of symptoms needed for patients to define themselves as in flare and to understand the support needs of RA men.

**Electronic supplementary material:**

The online version of this article (doi:10.1186/1471-2474-15-364) contains supplementary material, which is available to authorized users.

## Background

Rheumatoid arthritis (RA) is a chronic, systemic autoimmune disease, characterised by daily fluctuation of symptoms (e.g. pain, fatigue) and unpredictable disease flares [1, 2]. Early diagnosis and treatment is crucial [3] to avoid irreversible joint damage, potentially leading to permanent disability and increased personal impact [4]. It is possible that delayed treatment of RA flares may also cause irreversible damage due to prolonged periods of inflammation, although this has not yet been tested. However, little is known about patients’ motivations and barriers for seeking help for their RA flares.

There is a scarcity of research that addresses how patients specifically manage the symptoms and consequences of their RA flares. One qualitative study [5] within an ethnically diverse group of RA patients found that patients who did not believe there was a cause to their flares were more likely to use strategies aimed at managing their symptoms such as using analgesics or distraction techniques. In contrast, those who believed they could identify the cause of their flare were more likely to use strategies aimed at eliminating these perceived causes such as altering their diet [5]. A further qualitative study [6] with RA patients across five countries found that when in a flare, patients increase their usual level of self-management strategies by resting, pacing, applying heat or cold and escalating medications such as gluco-corticoids, often without seeking medical advice. Patients may be able to successfully self-manage early warnings of flare or divert minor flares, whereas unprovoked, persistent symptoms lead patients to redefine their flare as ‘uncontrollable’ [6]. Flare symptoms becoming uncontrollable (even with increased self-management strategies) and patients no longer being able to run their normal lives, prompts patients to seek professional help for their RA flare [6]. A further qualitative study [7] found that patients saw seeking medical help for their flares as a last resort once they had exhausted all self-management techniques. However, none of these studies explored patients’ help-seeking behaviours in depth, or whether there are common behaviour patterns [5–7]. Such understanding might help clinicians in supporting patients to self-manage their RA.

It is not only when patients experience a flare that they need to learn to manage their condition. Daily life with RA has been identified as full of ‘uncertainty’ relating to patients being uncertain about their own interpretations of their symptoms and whether they would be able to receive adequate help to master their disease and manage their everyday lives [8]. Uncertainty was also reported due to unpredictable exacerbations and remissions of disease, and its unpredictable long term course. Living with RA in day to day life also means having to relate, over time, to an increasingly non-compliant body; a body with RA does not move as desired [9].

It has been widely reported that living with RA impacts on hobbies, pastimes and with sexual activities [10–14]. The intrusiveness of RA has been reported to be greatest in areas of active recreation, work and health [15] with the intrusiveness of RA increasing as physical function worsens [16]. The overall effect of RA on individuals’ valued life activities appears to affect their psychological well-being. Loss of the ability to engage in recreational activities and social interactions in particular has been reported to significantly increase the risk of new onset depression [17].

A wide variety of self-management methods are recommended for people with RA and patients use technical aids, rest, exercise, heat packs, and joint protection on a daily basis [18]. Thus, it is necessary for patients to manage their symptoms every day even if they are not in a flare. Pacing and planning is also a recommended self-management technique for RA patients [19].

However, these studies were all conducted prior to the use of current, more aggressive treatment techniques, and despite these advances in clinical management [20, 21], only one recent qualitative study has explored how patients now experience and manage their RA on a day to day basis [7], this study reported patients move back and forth along a continuum (RA in the background versus the foreground), by balancing self-management of symptoms and demands of everyday life. [7] However, this was a qualitative study and therefore did not identify whether common patterns exist in patients’ experience of living daily with RA.

It is likely that individual patients experience life with RA and help-seeking for RA flares in different ways. Thus, producing overall consensus on these experiences creates the potential problem of providing a ‘bland generalisation’ of the issues [22] and masking individual differences, to provide an unrealistic averaging of beliefs [23]. It was therefore decided that Q-methodology would be an appropriate method to understand patients’ experiences of daily life and flare help-seeking as it produces Factors (groups of opinion) that each represent a different and independent understanding of the issue [24]. Identifying clearly distinct opinions and beliefs about the experiences of living with RA and of help-seeking behaviours would enable clinicians to tailor care to patients’ individual experiences and needs.

Thus, the current study aimed to identify clusters of consensus (rather than just overall consensus) on patients’ experiences of daily life with RA; and to identify clusters of consensus on RA patients’ barriers and prompts for help-seeking for flares [7].

## Methods

### Q-Methodology

**Figure 1:**
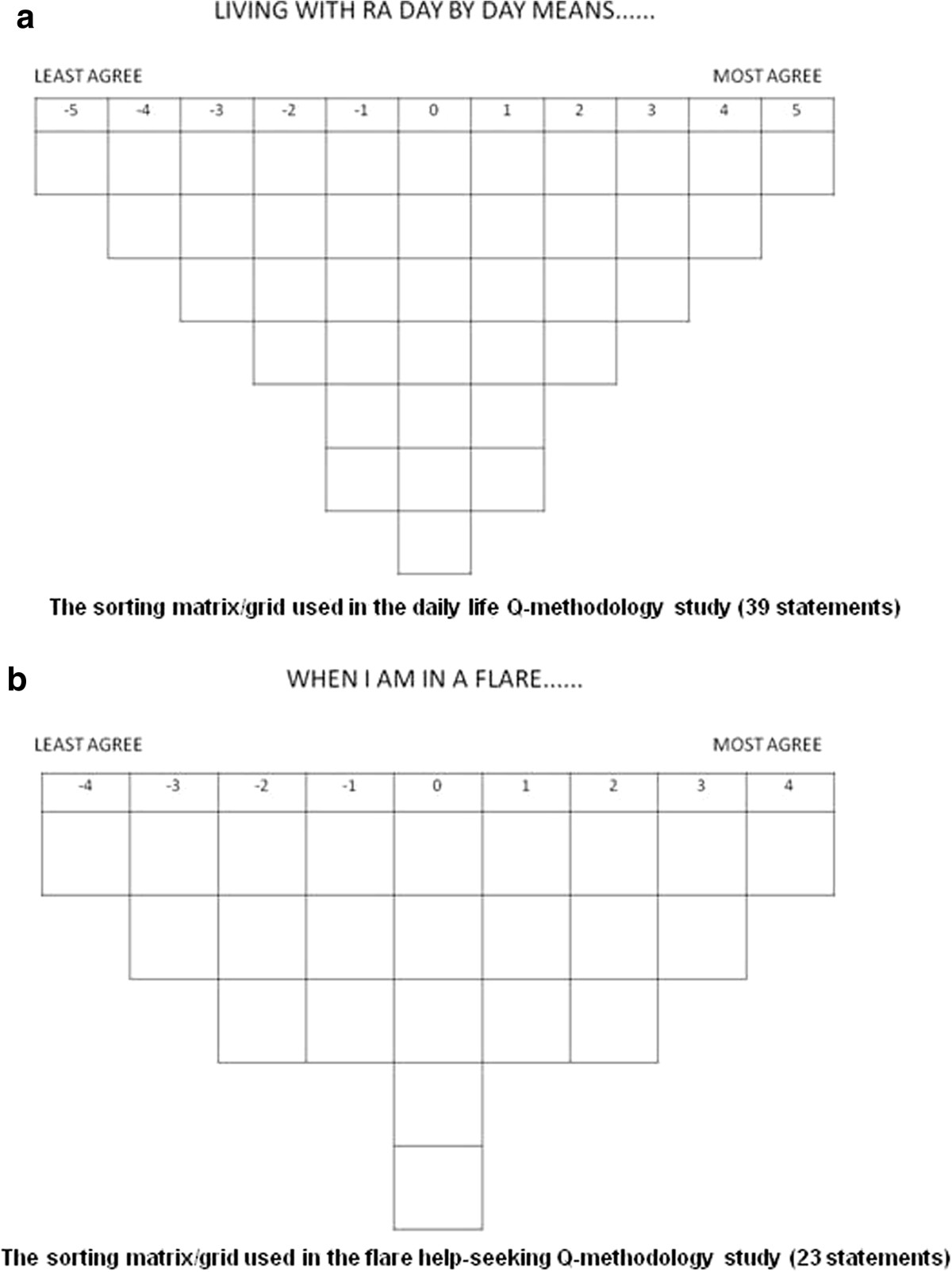
**The sorting matrices used in both Q-methodology studies. a**: The sorting matrix/grid used in the daily life Q-methodology study (39 statements). **b**: The sorting matrix/grid used in the flare help-seeking Q-methodology study (23 statements).

Q-methodology involves participants sorting a set of statements into the order of their agreement across a normal distribution grid. Q-methodology evolved from factor analytic theory to provide a way to reveal the subjectivity involved in any situation [25]. Q-methodology combines the strengths of both qualitative and quantitative research [26], providing a bridge between the two paradigms [27]. Q-methodology involves 3 stages: developing a set of statements to be sorted; sorting of those statements by participants along a continuum of agreement (Figure 1a,b); and analysis and interpretation of the sorted data [28, 29].

Participants are asked to rank-order statements of opinion (agree to disagree), which is known as ‘Q-sorting’. The statements are opinion only, not fact; Q-methodology assumes that opinions are subjective and can be shared, measured and compared [24, 30]. The sorting matrix provided for participants in Q-methodology (Figure 1a, b) forces the Q-sort into the shape of a quasi-normal distribution. There are fewer statements that can be placed at the extreme ends and more that are allowed to go into the middle area (the middle area represents almost neutral reaction). The symmetry and predetermined numbers of statements in each category facilitate the quantitative methods of correlation and factor analysis [30].

### Materials

#### Statements (Q-set)

To produce statements regarding RA patients’ experiences of daily life and their flare help-seeking behaviours, data from previous interviews with an earlier sample of 15 RA patients [7] were examined, alongside a comprehensive literature review and discussions with a patient partner (PR) [31]. After removing repeated or ambiguous items, 39 statements relating to daily life with RA (Q-study 1) and 23 statements relating to flare help-seeking (Q-study 2) were included. These were divided into two Q-studies as the issues were felt to be sufficiently distinct to require separate consideration by participants.

#### Q-sort grids and statement cards

One grid for each Q-study was designed, one containing 39 spaces for the daily life statements to be sorted onto (Figure 1a), the other containing 23 spaces for the flare statements to be sorted onto (Figure 1b). The grids were printed A1 size and laminated. Laminated cards were created with one statement on each card. Velcro was used to attach the individual cards to the grid. This ensured that the cards stayed in place during the sorting process, with the additional benefit of making the cards easier for RA patients to pick up from the table.

#### Participants

Patients attending outpatient clinics at one of three NHS Trusts, in different socio-economic locations and with different methods for accessing care, were invited to participate. Patients with confirmed RA [32] for >2 years (time to have adjusted to life with RA) and self-reported experience of a self-defined flare, were purposively sampled to reflect a range of age, gender, disease duration, disability and drug treatment. The same patients were invited to participate in Q-study 1 and 2. Thirty participants is considered an appropriate sample size to achieve stability in the resulting factor structure [33, 34].

Participants gave informed consent and ethics approval was granted (South West 4 Research Ethics Committee, 10/H0102/77). Thirty two of 72 patients (44%) agreed to participate but data were excluded from one who could not understand the task. In Q-study 1 (daily life), a second participant’s data were excluded as his wife completed the task on his behalf (n = 30). In Q-study 2 (flare help-seeking) this participant was included as he completed the task unaided, but two participants declined to complete Q-study 2 (no time, no recent flare experience) (n = 29).

#### Methods

**Figure 2:**
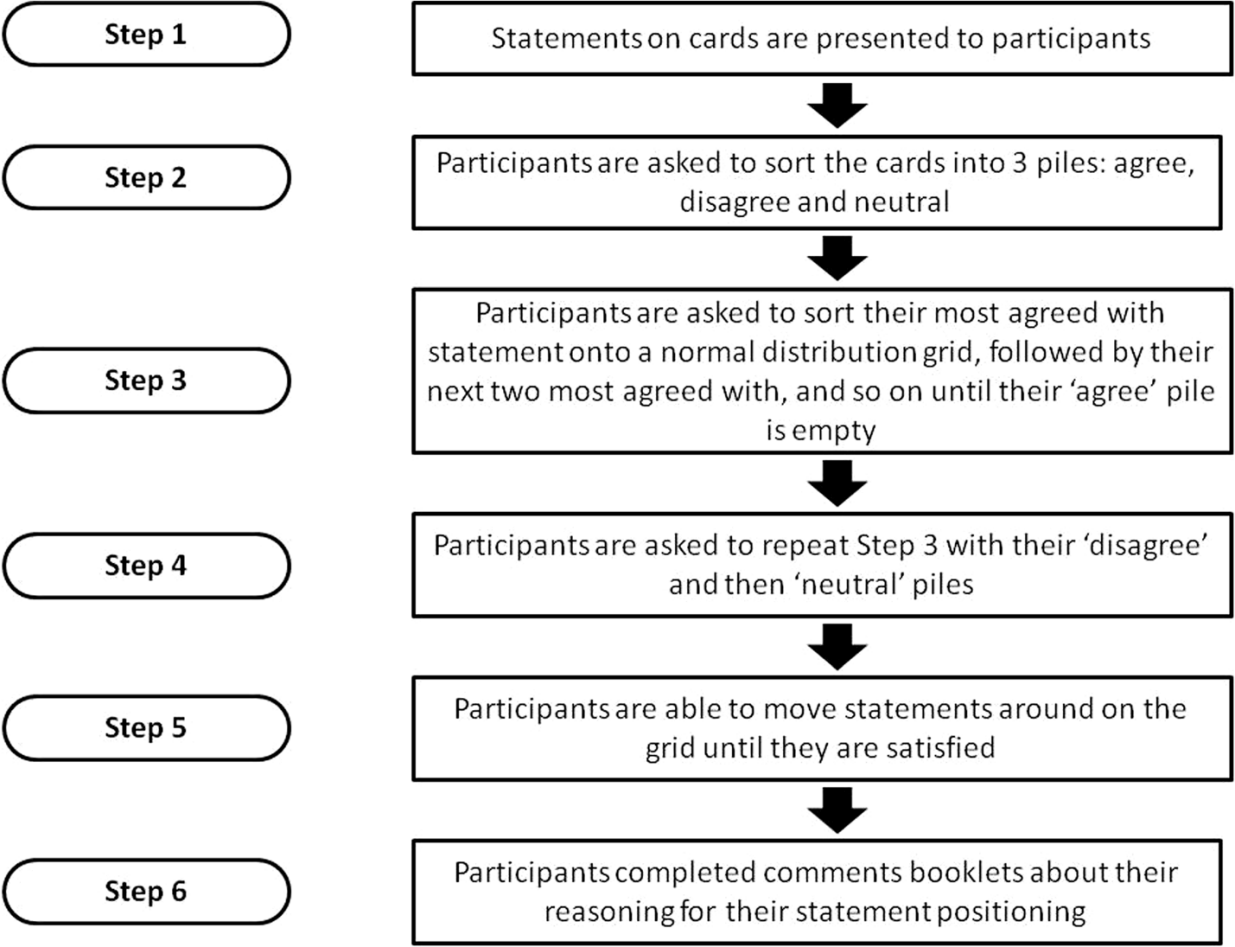
**Flow diagram showing the process of Q-sorting for participants.**

The study, lasting 1 to 1.5 hours, was conducted by an independent researcher (CF) in participants’ homes or non-clinical outpatient rooms (participants’ choice). Each participant was asked to divide the statements into three piles (agree, disagree and neutral). They were then asked to use these piles to help them arrange each numbered statement in approximate rank order of the degree to which they agreed with that statement *relative to the other statements*. Each numbered statement was placed in a single box on the Q-sort grid (Figure 1a,b), the total number of boxes reflecting the total number of statements. The grid box pattern allowed for the majority of statements to be agreed or disagreed with mildly or neutrally (with seven “0” boxes, and six “+1” or “-1” boxes each, for example), but only 1 statement could be placed in the highest agreement box (“+5”) or highest disagreement box (“-5”). Thus each participant’s opinions on the statements were forced into an “upside down triangle” (Figure 1a, b), generating a quasi-normal distribution of degrees of agreement with the statements [30]. The precise shape and limits of agreement/disagreement of this distribution (and the grid) is dependent on the number of statements (Figure 1a, 39 statements for Q-study 1 and 1b, 23 statements for Q-study 2).Once satisfied, participants commented on their statement positioning, particularly those statements placed in the extreme positions at each end of the grid, these qualitative comments were recorded in open-ended response booklets. Figure 2 provides a flow diagram of the process of this study.

### Analysis

Q-methodology combines qualitative and quantitative methods to produce a rounded interpretation of a single dataset (in contrast to a mixed-methods approach) [35]. Q-methodology analysis involves factor extraction, rotation and interpretation.

#### Factor extraction and rotation

Data were analysed using the PCQ software package [36]. In Q-methodology, participants are treated as variables and are intercorrelated and subjected to by-person factor analysis. The software searches for shared patterns (or sorting configurations) in the data and extracts portions of common variance (factors). For each Q-factor to be interpretable, an eigenvalue >1.0 (indicating factors are unlikely to have grouped participant views by chance), and ≥1 Q-sort loading significantly upon each factor alone is required [26]. Following extraction, the factors were rotated using orthogonal varimax rotation to ensure each Q-sort defined (has a high factor loading in relation to) only one of the study factors, so the overall solution maximises the amount of study variance explained [37]. For ease of interpretation it is standard Q-methodological practice to generate a single exemplary Q-sort by merging (according to a procedure of weighted averaging) the Q-sorts of all significantly loading participants: factor array [37].

#### Factor interpretation

For each factor, the open-ended (qualitative) comments from the factor exemplars (significantly loading participants) are combined with the factor array (single exemplary Q-sort produced by an average of all loading participants) to provide one Gestalt explanation of each factor [37, 38]. Statements scored similarly across all factors are considered to have reached consensus, for this paper only statements reaching consensus agreement ≥ +2 will be discussed, these only occurred in Q-study 2 (flare help-seeking).

## Results

### Presentation of results

Table 1 presents participant demographic data, along with the three factors identified for Q-study 1 (daily life) and two factors for Q-study 2 (flare help-seeking). These decisions were based on the solutions having: a maximal explained variance; a maximum number of Q-sorts loading significantly on one factor; all factors with eigenvalue >1.00; all factors containing statements distinguishing them from other factors; none of the sorts being confounded (i.e. significantly loading on more than one factor); and the researcher’s judgement. Table 2 provides a summary of characteristics of all factors presented. Tables 3 and 4 present the average rating (provided by the factor array) given by each of the three Daily Life factors and each of the two Flare Help-Seeking factors to each statement.

**Table 1:** **Participants and their factor loadings for the two Q-studies**

ID	Dis Dur (yrs)	HAQ	Pt Global	Med	In Flare?	Daily life factor	Daily life factor load	Flare factor	Flare factor load
**P1**	7	1.38	2.2	Biologic	No	A	0.48	X	0.86
**P2**	36	2.38	6.7	NSAIDs	No	A	0.70	Did not load	-
**P3**	23	2.63	2.6	Biologic	Yes	A	0.55	X	0.70
**P4**	16	2.63	2.3	Biologic	No	A	0.52	X	0.89
**P5**	32	2.25	2.6	Steroids	No	A	0.51	Did not complete	-
**P6**	31	1.50	4.0	Biologic	No	A	0.59	X	0.62
**P7**	14	1.38	1.5	Biologic	No	A	0.52	Did not complete	-
**P8**	Unknown	0.00	0.5	Biologic DMARDs	No	B	0.70	X	0.86
**P9**	40	2.25	6.9	DMARDs	No	B	-0.50	Y	-0.72
**P10**	27	0.50	4.3	DMARDs	No	B	-0.46	X	0.78
**P11**	4	1.63	4.7	DMARDs Steroids	No	B	-0.70	X	0.58
**P12**	2	2.00	5.4	Biologic DMARDs	No	B	-0.54	X	0.57
**P13**	5	0.63	6.7	DMARDs	Yes	B	-0.46	Y	-0.63
**P14**	25	2.13	5.5	Biologic Steroids	No	B	-0.54	X	0.60
**P15**	2	0.75	7.9	Steroids	No	B	-0.85	X	0.76
**P16**	17	2.14	1.0	Biologic DMARDs	No	B	-0.42	Did not load	-
**P17**	9	0.38	1.2	Biologic	Yes	C	-0.52	X	0.90
**P18**	6	0.00	0.3	Steroids	No	C	-0.42	Y	-0.71
**P19**	1	0.125	1.3	DMARDs	No	C	-0.67	Did not load	-
**P20**	16	0.88	3.4	Biologic	No	C	-0.44	X	0.78
**P21**	30	0.62	2.3	No medication	No	C	-0.63	Y	-0.60
**P22**	3	0.00	1.9	DMARDs	No	C	-0.48	Did not load	-
**P23**	4	1.25	1.5	Biologic	No	C	-0.59	X	0.67
**P24**	20	2.50	5.0	DMARDs	No	Did not complete	-	X	0.81
**P25**	30	0.88	0.3	Biologic	No	Did not load	-	X	0.55
**P26**	12	0.75	2.0	DMARDs	No	Did not load	-	X	0.76
**P27**	18	1.57	3.6	DMARDs	Yes	Did not load	-	Y	-0.70
**P28**	13	2.30	5.0	DMARDs	Yes	Did not load	-	Y	-0.59
**P29**	4	3.00	2.0	DMARDs Steroids	No	Did not load	-	Did not load	-
**P30**	11	1.13	1.7	DMARDs	No	Did not load	-	Did not load	-
**P31**	14	0.75	1.5	DMARDs	No	Did not load	-	Did not load	-
**Overall mean (SD)**	**15.7 (11.3)**	**1.4 (0.9)**	**3.2 (2.1)**						

*Dis Dur* = Disease Duration.

*HAQ* = Health Assessment Questionnaire score 0–3, high bad.

*Pt Global* = Disease activity score patient global measure 0–10, high bad.

Medication: *NSAIDs* = Non-steroidal anti-inflammatory *DMARDS* = Disease Modifying Anti-Rheumatic Drug.

**Table 2:** **Summary characteristics of factor groups (Mean, SD)**

Factor	Eigenvalue	% variance explained	Gender	Age (yrs)	Dis Dur (yrs)	HAQ	Pt Global	Summary
**Daily life study (n = 30)**	n/a	n/a	73.3% f	54.6 (11.8)	15.2 (11.3)	1.3 (0.9)	3.2 (2.2)	
			26.7% m					
**Daily Life Factor A (n = 7)**	3.29	11%	85.7% f	61.7 (10.3)	22.7 (10.8)	2.0 (0.6)	3.1 (0.7)	Patients ‘take active control’, they use effective self-management strategies, which they have developed through experience, they have become experts in managing their RA and they know what works for them. They take a practical approach to managing their RA, using preventative rather than crisis management techniques. They are predominantly female, older, with longer disease duration and more disabled.
			14.3% m					
**Daily Life Factor B (n = 9)**	3.97	13%	25.0% f	54.9 (7.1)	15.3 (14.3)	1.3 (0.9)	4.8 (2.5)	
			75.0% m					
**Factor B- (n = 8)**	n/a	n/a	37.5% f	55.5 (7.3)	15.3 (14.3)	1.5 (0.8)	5.3 (2.1)	These patients, mainly men, seem to prioritise their physical symptoms, reporting negative thoughts and emotions associated with their RA, and they cannot find self-management or coping strategies that work for them. They may therefore be experiencing a sense of hopelessness about their lives with RA. Despite having less disability than patients in Factor A, these patients seem to be in a ‘constant struggle’ with their RA.
			62.5% m					
**Factor B + (n = 1)**	n/a	n/a	Male	50.0 (n/a)	Not known	0.0 (n/a)	0.5 (n/a)	
**Daily Life Factor C (n = 7)**	2.67	9%	100% f	42.4 (11.2)	9.9 (10.2)	0.5 (0.5)	1.7 (1.0)	These patients, all female and younger on average than the patients in Factors A and B seem to ‘put RA in its place’ by prioritising their responsibilities above their RA. These patients have lower average HAQ and patient global scores than the other two daily life Factors, which may be the reason that they seem to experience less impact of their RA, but it may also be due to assigning less importance to their RA as a coping strategy.
			0% m					
**Flare Help-Seeking study (n = 29)**	n/a	n/a	72.4% f	54.6 (11.8)	15.2 (11.3)	1.3 (0.9)	3.2 (2.2)	
			27.6 m					
**Help-Seeking Factor X (n = 16)**	10.18	35%	68.8% f	54.8 (9.6)	15.2 (10.4)	1.4 (0.8)	3.3 (2.1)	These patients will not wait to seek help when they are in an RA flare. They make a ‘definite decision’ that their symptoms constitute a flare and that the medical team are there to help them.
			31.2% m					
**Help-Seeking Factor Y (n = 6)**	4.58	16%	66.7% f	50.5 (15.4)	18.7 (13.9)	1.2 (1.0)	4.1 (2.6)	These patients will wait to seek help for their RA flares, whilst going through a period of ‘cautious indecision’. These patients are indecisive as to whether their flare needs medical help as they hope it will go away on its own and are cautious of seeking help due to worries about wasting the medical team’s time and beliefs they should manage alone.
			33.3% m					

*Dis Dur* = Disease Duration *HAQ* = Health Assessment Questionnaire score 0–3, high bad.

*Pt Global* = Disease activity score patient global measure 0–10, high bad.

**Table 3:** **By-factor ranking of statements given in the Daily Life Q-study**

	Factor scores			
Living with RA day by day means…	Factor A+	Factor B+	Factor B-	Factor C-
**D1: I sometimes have to cancel plans due to my RA**	0	0	0	1
**D2:** Being more spontaneous with life	-1	+3	-3	+1
**D3:** Struggling to do certain things	+1	-2	+2	0
**D4:** Choosing my clothes according to how easy they will be to put on and take off	+1	+2	-2	-4
**D5:** I am unable to predict how bad my symptoms will be each day	-1	-3	+3	+1
**D6:** Taking longer to get things done than I think it should	+2	-2	+2	-2
**D7:** Finding different ways of doing the things I want to	+4	0	0	+2
**D8: Giving myself permission to leave a task half finished**	+1	0	0	-1
**D9:** Using tools or devices to aid me in daily tasks	+3	+1	-1	-5
**D10:** Finding a balance between asking for help and remaining independent	+2	-1	+1	0
**D11:** Doing what I want to do regardless of the consequences	-3	+2	-2	-1
**D12:** Choosing to prioritise pleasurable activities against chores	0	+2	-2	-2
**D13:** Not letting my RA get me down	+3	+3	-3	+4
**D14:** Relying on support from my family/friends/others	0	0	0	-3
**D15: Talking to other people with RA who are similar to me helps**	-1	+1	-1	-1
**D16:** Using alternative medicines/therapies to manage my RA symptoms	-4	+3	-3	0
**D17:** Distracting myself from my symptoms	+2	+1	-1	0
**D18:** Trying not to eat certain foods	-4	+4	-4	-3
**D19:** I am cautious of gaining weight and putting extra stress on my joints	-1	0	0	+3
**D20:** Exercising as much as I can	+1	+5	-5	+2
**D21:** Making small adjustments to my day or activities constantly because of my RA	+5	-1	+1	0
**D22:** Taking my medication exactly as prescribed	+3	+1	-1	+2
**D23:** Planning rest time into my week	+2	+1	-1	-3
**D24:** Feeling *lucky* in comparison to other people	-1	+2	-2	+5
**D25:** I have periods of being completely symptom free	-3	+4	-4	-2
**D26:** Dealing with the severity of my symptoms going up and down	+1	0	0	+1
**D27:** Getting frustrated due to my RA	-2	-3	+3	+2
**D28:** Feeling guilty about holding others back due to my RA	-2	-1	+1	-4
**D29:** Feeling that my body has let me down	-2	-1	+1	0
**D30:** Trying to forget that I have RA	0	+1	-1	+3
**D31:** Worrying because of my RA	-2	-3	+3	0
**D32:** Repetitive tasks make my RA symptoms worse	0	-2	+2	-1
**D33:** Being determined not to allow my RA to interfere with my responsibilities	+4	0	0	+4
**D34:** Being angry because of my RA	-5	-5	+5	-2
**D35:** Experiencing unexplainable fatigue/exhaustion daily	+1	-4	+4	+3
**D36:** Experiencing pain daily	0	-4	+4	+1
**D37:** Experiencing swelling daily	-1	-1	+1	-1
**D38:** Experiencing stiffness daily	0	-2	+2	+1
**D39:** Struggling to explain to family and friends what life is like for me	-3	-1	+1	-1

Statements highlighted in **bold** text show that consensus on the average score was reached across the factors Reading the table by column shows the comparative ranking of statements that characterise a particular factor. Reading the table by row shows the comparative ranking of a particular statement across factors.

**Table 4:** **By-factor ranking of statements given in the Flare Help-Seeking Q-study**

	Factor scores	
When I am in an RA flare....	Factor X	Factor Y-
**F1:** I feel the flare will last until I seek medical help	0	-3
**F2:** I will contact the medical team as soon as possible	+1	-3
**F3:** I am reluctant to seek medical help as I worry about wasting the rheumatology team’s time	-3	0
**F4: I am more reluctant to seek medical help when I think I’ve caused the flare**	-1	-2
**F5:** I am reluctant to seek medical help as I don’t think the Dr can do anything to help	-3	-1
**F6:** I am reluctant to seek medical help as I hope it’ll go away on its own	-1	+4
**F7: I avoid seeking medical help as I don’t like taking drugs**	-2	-2
**F8:** I seek help from the medical team once flare starts to affect my quality of life too much	+4	+1
**F9:** I know I don’t have to manage my flare alone	+2	+1
**F10:** Easy access to the medical team is part of my decision to seek help for my flare	+1	0
**F11:** A loved one tells me I ought to seek medical help	0	+2
**F12:** I don’t like admitting that I need to ask for help	-1	+2
**F13: I am reluctant to seek medical help as I don’t get on well with my rheumatology team**	-4	-4
**F14:** I manage my symptoms until the flare stops	0	+1
**F15:** I seek help from the medical team as I worry about long term damage to my joints	+1	-1
**F16: I seek help from the medical team when the pain becomes too intense**	+3	+3
**F17:** I wait until my next scheduled appointment with the rheumatologist before seeking help	-2	-1
**F18:** I am reluctant to seek medical help as I don’t want to waste my own time	-2	0
**F19:** I seek help from the medical team when I know my flare needs to be controlled by new medication	+2	-2
**F20: I control my flare symptoms with medication before contacting the medical team**	0	0
**F21: I seek help from the medical team when my symptoms become uncontrollable**	+3	+3
**F22: I seek help from the medical team when the flare has gone on longer than I expected**	+2	+2
**F23: I wait until I have more than one flare symptom before seeking medical help**	0	0

Statements highlighted in **bold** text show that consensus on the average score was reached across the factors.

Reading the table by column shows the comparative ranking of statements that characterise a particular factor. Reading the table by row shows the comparative ranking of a particular statement across factors.

### Daily life (Q-study 1)

Thirty patients (22 women) participated in the daily life Q-study (mean age: 54.6 yrs, SD: 11.8, mean disease duration: 15.2 yrs, SD: 11.3) (Table 1).Three factors were extracted and rotated, explaining 33% of the variance and accounting for 23 of the 30 participants. Participant loading of ≤ ±0.41 reached significance at *p <* 0.01, indicating that each loading participant closely exemplifies the factor they load onto [37].

#### Interpretation of Factor A: Taking active control: “Just a fact of life” (P2)

Seven participants significantly loaded onto this factor. They are predominantly female, older, diagnosed with RA longer and more disabled than those loading onto the other two daily life factors (Table 2).

Factor A exemplars ‘take active control’ of their RA through effective self-management techniques, such as micromanaging their daily lives due to RA (Table 3, statement D21: opinion +5), as Participant 1 commented:

*“This is important as you need to be able to do this to manage your RA effectively” (P1)*

They work around their RA and don’t let it interfere with their responsibilities [D7: +4; D33: +4], they take responsibility for managing their disease [D22: +3; D11: -3]:

*“It’s [taking medication exactly as prescribed] important and part of my routine”* (P5)

*“You can’t just do this [what you like regardless of the consequences], people that do this make me angry. You can’t expect the doctors to help you if you don’t help yourself”* (P4)

Factor A exemplars know when to accept help from tools and devices or other people [D9: +3; D10 + 2] and have learned which self-management techniques do not work for them [avoiding certain foods: D18: -4; alternative medicines: D16: -4] (*“I don’t find they make any difference*”: P2).

Although still experiencing symptoms daily [D25: -3; D36: 0; D35: +1; D37: -1; D38: 0], these patients experience less impact from their RA due to attaching less importance to their symptoms:

*“You get used to your symptoms, but because of the disability you’re never symptom free”* (P7)

*“Just a fact of life, they’re sorted lower as they’re not as interesting as the other statements”* (P2)

Thus they do not report low mood due to RA [D31: -2; D34: -5; D13: +3] and will not say that their body has let them down [D29: -2] as “*that’s a negative way of thinking”* (P7).

#### Interpretation of Factor B: Constant struggle: “It gets me down every single day” (P15) (B-) and Feeling good: “anti-TNF has kept me working” (P8) (B+)

Nine participants load significantly on this factor. Eight load negatively and one loads positively. Thus, this is a ‘bipolar’ factor, meaning that two opposed viewpoints are expressed, which each have a factor array that is the ‘mirror-image’ of the other, thus only the negative loading (Factor B-) will be presented. Negatively loading participants are predominantly male (71%), in comparison to the other two factors and overall study population (26%) (Table 2).

Factor B- exemplars appear to struggle with their RA, they report never being completely symptom free [Table 3, D25: -4]. They experience unpredictable symptoms daily [D5: +3] with pain and fatigue being particularly problematic [D36: +4; 35: +4]. These patients get angry [D34: +5] and worry [D31: +3] because of their RA:

*“I get very frustrated with it, the problem is then I get irritated and take it out on the wife”* (P9)

*“It [RA] gets me down every single day”* (P15) [D13: -3]

Factor B- exemplars seem to struggle to find a way of managing their RA. They will not avoid certain foods [D18: -4] as they *“don’t know what to avoid”* (P12) nor try alternative medicines [D16: -3] because *“they’re a waste of time and money”* (P14). They feel unable to exercise [D20: -5], be spontaneous [D2: -3], or prioritise pleasurable activities over chores [D12: -2]. They struggle to do certain things [D3: +2] and find it takes longer to do things than they think it should [D6: +2], which they find frustrating [D27: +3]:

*“Very frustrating for me as I used to do things quickly”* (P11)

*“I always did things quickly, I didn’t ever sit around. I find the less I do, the less I want to do and I don’t want that. I find it very frustrating”* (P14)

The positive loading participant (P8) holds the polar opposite viewpoint to that presented above.

#### Interpretation of Factor C: Keeping RA in its place: “It’s a very small part of you” (P22)

Seven participants load significantly onto this factor; all female, younger, diagnosed for less time and report less disability in comparison to the other factors (Table 2). Five had dependent children.

Factor C exemplars prioritise their responsibilities above their RA [Table 3, D33: -4] and find it necessary to ‘keep RA in its place’ by finding different ways of doing things [D7: +2]. However, they feel too busy to plan rest time [D23: -3]:

*“I’ve lived with it for a long time now, I’m not going to allow it to ruin my life, it’s not fair on the children. I’ll do what I can for the children, even if I suffer for it”* (P20)

*“Not going to happen [rest time]. I run my own business and have two small children”* (P20)

These patients experience very little impact of RA on their daily activities [D6: -2]. They are also able to forget about their RA [D30: +3], including when choosing food [D18: -3] or clothes [D18: -3]:

*“This works. I am able to forget about it a lot of the time. My Consultant said to me ‘This is you [draws circle] and this is your RA [draws much smaller circle]’. It’s a very small part of you, and I believe that”* (P22)

*“I never think of my RA when I’m shopping for clothes, I just wear what I like”* (P18)

These patients do not get angry [D34: -2] nor allow RA to get them down [D13: +4]. They feel particularly lucky in comparison to other people [D24: +5]:

*“Definitely – most important [statement]. RA runs in the family, so I’ve seen relatives in wheelchairs and very unwell with it and you see other people in clinic. I look and feel so well with it.”* (P17)

They are self-sufficient; they *“don’t hold others back”* (P23) [D28: -4] and “*don’t ask for help”* (P19) from family and friends [D14: -3] nor use tools to aid them [D9: -5].

However, they are concerned about gaining weight and putting extra stress on their joints [D19: +3]; it’s *“a constant worry”* (P19) and therefore try to exercise [D20: +2].

### Flare help-seeking (Q-study 2)

Twenty nine patients (21 women) participated in the flare help-seeking Q-study (Table 1). Two factors were extracted and rotated, explaining 51% of the variance and accounting for 22 of the 29 participants. A participant loading of ≥ ±0.54 or above reached significance at *p* <0.01.

#### Consensus statements

Of the statements scored similarly by both factors (highlighted **bold** in Table 4), those reaching consensus agreement ≥ +2 related to tipping points for help-seeking: flare lasting longer than expected [F22: +2, +2], pain becoming too intense [F16: +3, +3] and symptoms becoming uncontrollable [F21: +3, +3]:

*“When I just don’t know what to do anymore”* (P25)

#### Interpretation of Factor X: Definite decisions: “It won’t go away, so I won’t wait” (P10)

Sixteen participants significantly loaded onto this factor. They are predominantly female (11/16) (Table 2) and taking biologic therapies (11/16).

Factor X exemplars will contact the medical team as soon as possible when in flare [F2: +1] and won’t wait for their next scheduled appointment [F17: -2]. They believe the medical team will be able to help them [F5: -3] and doubt their flare will go away without help [F6: -1]:

*“I’ll contact the next day and I get seen very quickly”* (P11)

*“Minor aches and pains go away on their own, flare-ups don’t”* (P8)

They don’t worry about wasting their own [F18: -2] or the rheumatology team’s time [F03: -3]: *“that’s what they’re there for”* (P17).

Tipping points for help-seeking specific to Factor X exemplars are worries about long term joint damage [F15: +1], believing they need a change in medication [F19: +2] and flare beginning to affect their quality of life [F8: +4].

#### Interpretation of Factor Y: Cautious indecision: “Lying down and not moving until it goes”

Six participants significantly loaded onto this factor. They are predominantly female (4/6) (Table 2) and none were using biologic therapies.

Factor Y exemplars appear both cautious and indecisive in seeking help and will not contact the medical team urgently when they are in flare [F2: -3]. They are reluctant to seek help and hope the flare will go away on its own [F6: +4; F1: -3]. They worry more than Factor X exemplars about wasting the rheumatology team’s time [F3: 0], which appears to be due to beliefs they should manage alone. A statement scored neutrally can indicate cautious agreement, and these participants’ comments support this:

*“I was brought up to be self sufficient and not run for help, I know that I should know better, but it’s ingrained”* (P27)

*“I do worry about this [wasting the rheumatology team’s time], even though I shouldn’t. I went to the Doctor and had to keep going back for help and it wasn’t getting any better – but it wasn’t getting any worse. So I thought, they know how bad it is, they don’t need to see me unless it gets worse, so I left it”* (P9)

These patients will try and manage their symptoms until the flare stops [F14: +1], and will wait until they are prompted by a loved one to seek help [F11: +2].

## Discussion

This study indicates that in daily life some RA patients are able to keep a balance through effective self-management strategies, some (predominantly men) struggle to cope and others (predominantly younger women) put their life and/or work responsibilities before their RA self-management. Patients will seek help for flares due to pain intensity, flare longevity and uncontrollable symptoms. However, whilst some patients will quickly define their symptoms as a flare and make a ‘definite decision’ to seek help, others are indecisive as to whether their symptoms constitute a flare and are cautious of seeking help due to beliefs they should manage alone.

This study suggests disease severity, importance and self-management affect the daily personal impact of RA and these can mediate each other. These may be the first data to support the proposal of such an ‘impact triad’ [39]. Furthermore, personal characteristics or roles (e.g. gender) may play an important role. The younger women in the daily life Q-study reported not allowing RA to impact on their lives. They were more recently diagnosed, which may explain their lower disability scores, and why they report less impact from RA. However, the results also suggest these women will seemingly prioritise their responsibilities over well-being and may be minimising the impact of RA as a coping strategy [40]. Furthermore, patients typified by Factor B (mainly men) attach more importance to their symptoms and seem unable to find self-management strategies that work for them, thus despite having less disability than Factor A-loading participants they experience a greater impact from their symptoms. The finding that men may be struggling to cope with RA provide further data to support a previous small qualitative study [41], which found that men and women had different coping needs. It has been proposed that a new health strategy is needed, that considers men’s specific needs [42]. The current study supports this and suggests further investigation into the experiences and support needs of men with RA.

The flare help-seeking Q-study consensus supports previous research [6, 7] that patients seek help when they are certain they are in flare, but feel unable to control it. This study also provides novel data, that patients are also prompted to seek help by pain intensity and flare longevity and although some patients will seek help quickly (‘definite decision’), without ‘trying to regain control’, others will delay help-seeking (‘cautious indecision’) whilst ‘trying to make sense of fluctuations’ and ‘trying to regain control’, often due to beliefs they should manage alone.

The majority of patients who reported rapid help-seeking were taking biologic therapies, whereas those who reported waiting were not. It is possible that patients on biologics have a different experience of care due to regular contact with the team for repeated medication reviews. Patients not prescribed biologics may consider their symptoms invalid, due to not being offered this treatment, which they worry about failing to ‘qualify’ for [43].

This study asked patients’ perceptions of whether they were in flare at baseline rather than using an objective measure, therefore it is unknown whether patients were in an inflammatory flare or not. Patients were not asked to provide their definition of flare, but this was a pragmatic study, and the current lack of consensus of flare definition may already pose a clinical problem [6, 44]. A limitation of Q-methodology is that participants are required to sort predetermined statements and are therefore constrained by the method [45]. However, the statements to be sorted came from recent interviews with RA patients [7] and the literature and therefore included a wide range of relevant opinions, they were also reviewed by a patient research partner (PR). In addition, patients were sampled from three NHS Trusts, all with different methods for accessing care and from different socio-economic areas within the UK.

Clinicians should also be aware that there are at least four ways in which patients experience life with RA: ‘Feeling Good’, ‘Taking Active Control’, ‘Keeping RA in its Place’ and ‘Constant Struggle’ and these patients would require different levels and types of support. Some patients manage well due to low symptoms or expert self-management and therefore appear to need little intervention from the medical team. One group of patients of particular note are male RA patients, who appear to have a more negative experience of RA than female patients. These male patients focus on their symptoms, experience negative thoughts and feelings and do not seem able to identify effective self-management techniques. This has an important clinical implication for the way in which men with RA are supported. These male patients may benefit from a tailored intervention, but their specific support needs require further research before an effective intervention can be designed.

The findings from this study also indicate that some patients would benefit from patient education programmes to ensure they are aware of when and how to seek help for their RA flares. Cognitive Behavioural Therapies (CBT) [46] are growing in application within the UK health care system. In RA positive results have been found for tailored CBT interventions in the reduction of depression, helplessness, fatigue and enhanced the use of active coping strategies [47–49]. Thus based on these findings, one subset of patients may benefit from clinicians addressing psychological issues to empower them to seek help.

## Conclusions

Daily life with RA has less impact on some patients than others, which can be due to less disease severity, expert self-management or attaching less importance to their RA. However, RA has a larger impact on other patients, the majority of whom seem to be male, due to concentrating on physical symptoms and rejecting self-management techniques.

In an RA flare some patients will seek help quickly, whilst others will wait due to beliefs that they should manage alone. Patients will seek help when their pain is too intense, their flare has gone on longer than expected and they are no longer able to control their flare. Patients may therefore see medical help-seeking as a last resort, when they are no longer able to cope alone. Further research is needed firstly to quantify the nature and level of symptoms still experienced in daily life while on current treatments; and secondly in understanding men’s experiences of RA and their support needs.

## Authors’ original submitted files for images

Below are the links to the authors’ original submitted files for images. Authors’ original file for figure 1 Authors’ original file for figure 2
