# Supplementary figures and images for: A Q-methodology study of flare help-seeking behaviours and different experiences of daily life in rheumatoid arthritis
Source: BMC Musculoskelet Disord. 2014 Nov 1;15:364. doi: 10.1186/1471-2474-15-364 (PMC4223853; doi:10.1186/1471-2474-15-364)

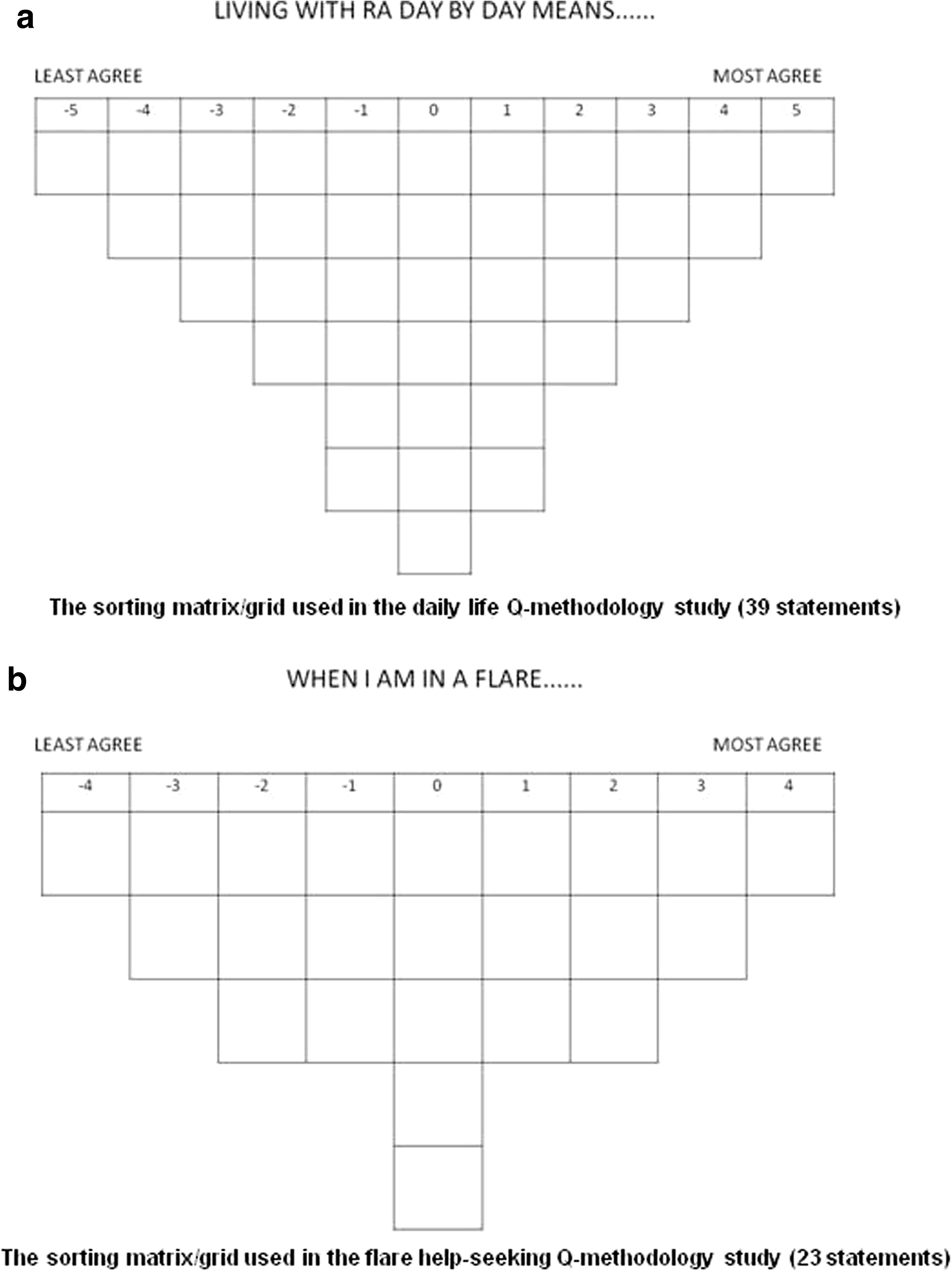

Supplement: Supplementary file 1 — Authors’ original file for figure 1 [file 12891_2014_2296_MOESM1_ESM.tif]

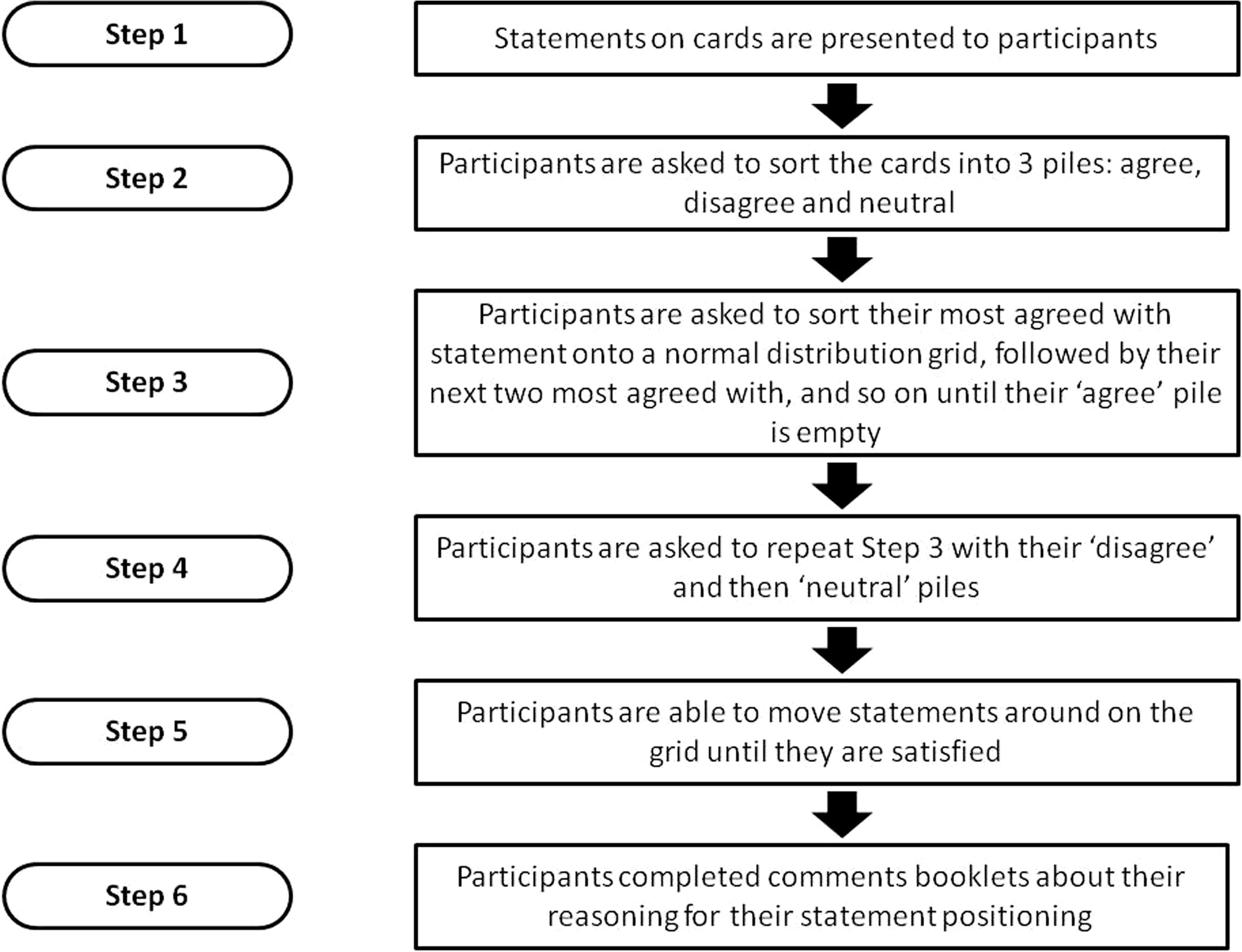

Supplement: Supplementary file 2 — Authors’ original file for figure 2 [file 12891_2014_2296_MOESM2_ESM.tif]
